# Examining the relationship between foreign language anxiety and foreign language enjoyment from the perspective of positive psychology

**DOI:** 10.3389/fpsyg.2025.1594282

**Published:** 2025-04-25

**Authors:** Qiangfu Yu

**Affiliations:** Faculty of Humanities and Foreign Languages, Xi’an University of Technology, Xi’an, China

**Keywords:** foreign language anxiety, foreign language enjoyment, foreign language learning, foreign language learners, academic achievements, positive psychology

## Abstract

Negative emotions represented by foreign language anxiety (FLA) have long been an important topic in the study of second language acquisition (SLA), while the influence of positive emotions on foreign language learning (FLL) has long been neglected. Since Positive Psychology (PP) was introduced into the field of SLA, research on learners’ positive and negative emotions in SLA based on the perspective of PP has begun to flourish. Particularly, the correlation between foreign language enjoyment (FLE) and FLA has become a hot topic. Based on the analysis of the literature on FLA and FLE, it was found that the research on FLA and FLE mainly focuses on the following categories: correlation between FLA and FLE, influencing factors of FLA and FLE, correlation of FLA and FLE with academic achievements, and dynamicity of FLA and FLE. This study summarized the major findings in the four research hotspots and concluded with some research lacunae and possible directions for future research on FLA and FLE from the perspective of PP.

## Introduction

Since the 1970s, emotions have been a hot topic in the field of SLA. However, a plethora of studies focus on the negative emotions, among which FLA is the most studied emotion in the past few decades (Scovel, 1978; Horwitz, 2001, 2010; MacIntyre, 2017). In recent years, with the introduction of PP into the field of SLA (Mercer and MacIntyre, 2014), positive emotions represented by FLE has gradually attracted the attention of researchers (Dewaele and MacIntyre, 2014, 2016, 2019; Dewaele et al., 2016; Dewaele et al., 2017; Mierzwa, 2020; Özer and Altay, 2021). PP favors a more holistic view of emotions of human beings, which means that instead of focusing solely on negative emotions, such as FLA, learners’ positive emotions, such as FLE, are considered equally (Dewaele and MacIntyre, 2014; Dewaele et al., 2016; Saito et al., 2018). Therefore, research on the positive and negative emotions of learners in SLA from the perspective of PP is flourishing, among which the relationship between FLA and FLE and its related variables has become a hot research topic (Dewaele and MacIntyre, 2014, 2016; Dewaele and Dewaele, 2017, 2020; Dewaele et al., 2017, 2019; Jiang and Dewaele, 2019; Li, 2020a; Li et al., 2021; Li and Han, 2022). However, as a relatively new research topic in SLA, studies on FLA and FLE combined are relatively rare, compared with studies on the single emotion of FLA or FLE. The present study aims to investigate the research status of FLA and FLE in the past decade by analyzing the research hotspots, and to provide some constructive suggestions for future research, based on the research lacunae of the current research.

## Literature review

### FLA

Anxiety is a subjective feeling of tension and worry associated with the arousal of the autonomic nervous system (Spielberger et al., 1983), a fear without an identifiable threat (Ohman, 2008). Boudreau et al. (2018) pointed out that anxiety is essentially a diffused form of fear. FLA is considered to be anxiety experienced by individuals in the course of learning a foreign language (FL) in a classroom or in any situation in which a FL is used (Gardner and MacIntyre, 1993). Horwitz et al. (1986) defined FLA as a unique complex structure of self-cognition, belief, feeling and behavior related to classroom learning in the process of FLL, and designed the Foreign Language Classroom Anxiety Scale (FLCAS) to help language teachers and scholars better understand students’ FLA. Gregersen and MacIntyre (2014) described FLA as a negative emotion of worry and anxiety caused by learning and using a second language in a classroom that requires self-expression. Overall, FLA is a kind of negative emotion in which learners feel frustrated, fearful and worried about FLL, including a series of anxiety related to specific language skills (Horwitz, 2016), and it is one of the strongest predictors of success or failure in FLL (Horwitz, 2010).

Studies on FLA mostly focus on the development and measurement of FLA scales (Horwitz et al., 1986; Wang, 2003; Dewaele and MacIntyre, 2014; Dewaele and Alfawzan, 2018; Dewaele et al., 2019a; Du, 2019; Jiang and Dewaele, 2019), the correlation between FLA and academic achievement (Gardner, 1985; Horwitz et al., 1986; Gardner and MacIntyre, 1993; Aida, 1994; Elkhafaifi, 2005; Horwitz, 2010; Dewaele and MacIntyre, 2014; MacIntyre, 2017; Mierzwa, 2020; Dong, 2021) and influencing factors of FLA (Dewaele, 2013; Gregersen and MacIntyre, 2014; Oxford, 2017; MacIntyre, 2017; Dewaele et al., 2019a; Dewaele and MacIntyre, 2019; Jiang and Dewaele, 2019).

### FLE

Enjoyment is regarded as an individual’s sense of achievement and satisfaction from the process and result(s) of some activity (Csikszentmihalyi, 1990; Ainley and Hidi, 2013). Martin Seligman (2012), the founder of PP, proposed PERMA (positive emotion, engagement, relationships, meaning, and accomplishment) model from the perspective of PP. Enjoyment constitutes a positive psychological experience characterized by an elevated emotional state that emerges when individuals transcend homeostatic equilibrium. This adaptive process facilitates personal development and sustains well-being, distinguishing it from pleasure, a more immediate gratification mechanism that serves to fulfill biological drives such as hunger reduction or physical comfort (Seligman and Csikszentmihalyi, 2000). Dewaele and MacIntyre (2016) conceptualized enjoyment as a multifaceted emotional state arising from the dynamic interplay between task challenge and perceived competence, where individuals derive intrinsic satisfaction through accomplishing challengeable tasks. Succinctly put‌, rooted in PP, enjoyment serves as a foundational catalyst for personal growth, thereby providing theoretical grounding to integrate PP theories into FLL research.

FLE extends MacIntyre and Gregersen’s (2012) application of PP principles to emotion regulation in second language acquisition. FLE, a relatively new concept, was proposed for the first time by Dewaele and MacIntyre (2014), who held that FLE can create a pleasant and reassuring psychological atmosphere for learners to explore unacquainted languages and cultures, thus promoting their FLL. Dewaele and MacIntyre (2016) further clarified that FLE is not only a positive emotion that promotes FLL, but also can alleviate the persistent effects of negative emotions by improving attention and awareness of FL input, and improve individuals’ resilience in coping with FLL difficulties. Overall, FLE is a complex and relatively stable emotion (Boudreau et al., 2018), which enables learners to form good interpersonal relationships in FLL, fuel the FLL process (Dewaele, 2022), and make continuous progress toward their learning goals (Chen Wen et al., 2021).

Since Dewaele and MacIntyre (2014) first proposed the concept of FLE, many scholars have begun to shift their attention from the study of negative emotions represented by FLA to the study of positive emotions represented by FLE from the perspective of PP. Most studies on FLE focus on the development and measurement of FLE scales (Dewaele and MacIntyre, 2014, 2016; Dewaele and Dewaele, 2017; Dewaele and Alfawzan, 2018; Li et al., 2018; Jin and Zhang, 2018, 2019; Dewaele et al., 2019a, 2019b), the relationship between FLE and academic performance/ achievements (Piechurska-Kuciel, 2017; Khajavy et al., 2017; Saito et al., 2018; Mierzwa, 2020; Dewaele and Alfawzan, 2018; Botes et al., 2021), influencing factors of FLE (Dewaele and MacIntyre, 2014, 2019; Dewaele and Dewaele, 2017; Dewaele et al., 2017, 2019; Dewaele and Alfawzan, 2018; Moskowitz and Dewaele, 2019; Jiang, 2020).

## Methods

Firstly, previous studies on FLA and FLE published in international journals were searched in databases of Web of Science (WOS) in February, 2025. The author used the following searching parameters to conduct the search for previous studies: (((TS = (anxiety)) AND TS = (enjoyment)) AND TS = (foreign language)) AND PY = (2014–2024) and (((TS = (anxiety)) AND TS = (enjoyment)) AND TS = (second language)) AND PY = (2014–2024). Secondly, studies published in Chinese journals were searched in China National Knowledge Infrastructure (CNKI) database in February, 2025. The author used the Chinese counterparts of the searching parameters mentioned above and extracted the studies on FLA and FLE in Chinese Social Sciences Citation Index (CSSCI) source journals.

**Figure 1:**
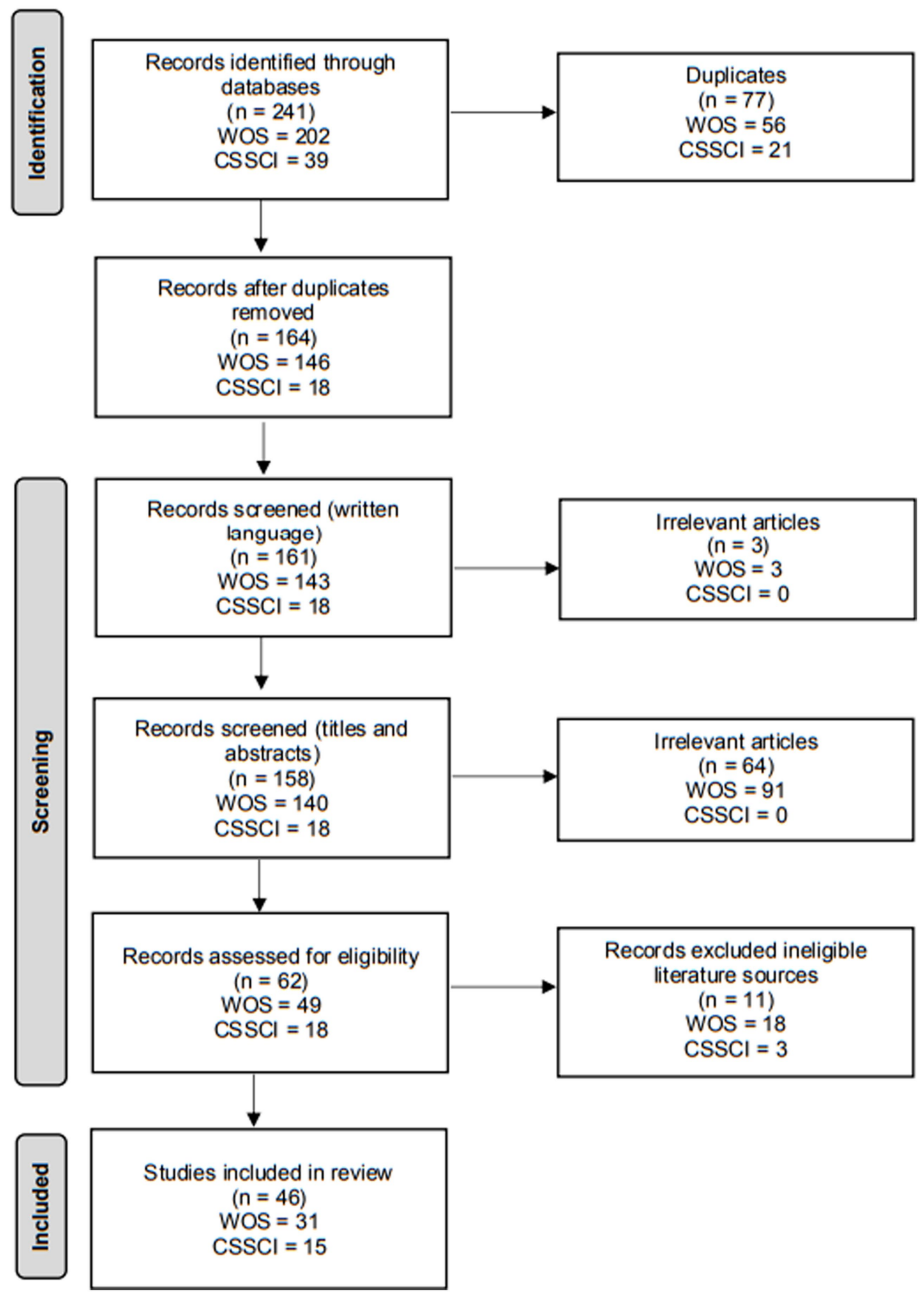
Flow diagram illustrating the process of article selection.

Studies were eligible for inclusion if they focused on FLA and FLE and if the relevant key words (e.g., anxiety, enjoyment, foreign language, second language) appeared in the titles or abstracts. We included all studies, both speculative or theoretical research design, empirical research design, qualitative and quantitative, in this review. Only CSSCI articles published in Chinese and WOS articles published in English were included. Then, the retrieved studies were evaluated for the eligibility by scanning and analyzing the full texts, and only studies focusing FLA and FLE were eligible for inclusion in this review. Conference proceedings, editorials, book reviews and review articles were outside the scope of the study and therefore excluded from the data collection. The inclusion and exclusion criteria as well as the selection procedure are summarized in Figure 1.

## Results and discussion

As a result, 31 papers were retrieved from Web of Science, and 15 from CSSCI. Based on iterative reading and analysis of the 46 retrieved studies, the hotspots of research on FLA and FLE can be summarized under the following categories.

### Correlation between FLA and FLE

From the perspective of PP, FLE aligns closely with the field’s emphasis on strengths and well-being cultivating while FLA reflects challenges to psychological resilience, highlighting the complex interplay between FLA and FLE. Many studies retrieved focus on the correlation between the two significant affective factors. Some scholars believed that there was a negative correlation between FLA and FLE (Maican and Cocorada, 2021). Dewaele and MacIntyre (2014) investigated the status quo of FLA and FLE among 1746 learners from different ages around the world and revealed the negative correlation between FLE and FLA, indicating that although FLE and FLA were related to a certain extent, they were independent in essence, which is of landmark significance to the development of PP. In a follow-up study, Dewaele and MacIntyre (2016) further explored the negative correlation between FLE and FLA, and verified the mutual independence of the two. Their findings resonate with PP’s distinction between reducing pathology (e.g., anxiety) and cultivating well-being (e.g., enjoyment), suggesting dual pathways for optimizing FLL outcomes.

Notwithstanding many researchers championed the negative correlation between FLA and FLE, they were somewhat divided on the degree of negative correlation between FLA and FLE. Dewaele and Dewaele (2017) found that there was a weak negative correlation between FLA and FLE among 189 FL learners in two London secondary schools. Dewaele et al. (2017) also found a low negative correlation between FLA and FLE. However, Dewaele and MacIntyre (2019) found a moderate negative correlation between FLA and FLE among 750 FL learners. Similarly, Dewaele et al. (2019a) found a moderate negative correlation between FLA and FLE through a questionnaire survey among 210 Spanish learners of English as a FL. Notwithstanding, Shirvan and Taherian (2021) analyzed FLA and FLE among 371 undergraduates by using the potential growth curve model and triangular data collection method, and found that students’ FLE level increased and FLA level decreased during the study period, and that FLA and FLE were significantly negatively correlated, which aligns with PP’s upward spiral model, where momentary positive emotions accumulate to produce enduring resilience (Garland et al., 2010).‌ Fang and Tang (2021) also found a significant negative correlation between FLA and FLE.

Contrary to dominant findings, some scholars also believed that there was a positive correlation between FLA and FLE. For example, Dewaele et al. (2019b) found a weak positive correlation between FLA and FLE among 592 undergraduates and middle school students in Kazakhstan who spoke Turkish as a FL, which reflects moderate anxiety might coexist with enjoyment during optimally challenging tasks, a phenomenon requiring nuanced PP frameworks to interpret.

### Influencing factors of FLA and FLE

Dewaele and MacIntyre (2014) investigated FLA and FLE among 1746 learners of different ages around the globe and found the participants’ age, gender, cultural background, FL level, education level and the number of FLs being learned were related to FLA and FLE. Based on the research above, Dewaele et al. (2016) specifically studied gender differences in FLA and FLE, and found that females experienced more emotions in FL classes, and their FLA and FLE were much higher than those of males. However, Wang (2023) found gender did not affect FLA and FLE of Chinese EFL learners. Dewaele et al. (2017) found that although the age of the participants had no correlation with FLA, it was positively correlated with FLE, and the girls had higher levels of FLA and FLE. The study also revealed FLE was related to positive classroom relationships and FL, while lower FLA correlated with the attitudes toward FLL and peer support, which mirrors PP’s broaden-and-build theory. Besides, Zhao and Zhang (2021) found no significant difference in FLA and FLE of English majors in different grades, and the students’ FLA and FLE came from classroom activities, teachers’ teaching methods, classmates’ support and personal progress in English learning.

### Correlation of FLA and FLE with academic achievements

Since FLA and FLE have an important effect on learning performance, they should be studied together from the perspective of PP to better explain learners’ emotional experience (Özer and Altay, 2021). Dewey et al. (2018) investigated 36 overseas students and found a negative correlation between language proficiency and FLA and a positive correlation between language proficiency and FLE. Li et al. (2020) explored FLA/FLE effects among 1,307 Chinese students, finding FLA negatively predicted learning performance while FLE positively predicted it. Similarly, Mierzwa (2020) found that FLE was positively correlated with academic performance, while FLA was negatively correlated with academic performance.

Dewaele and Alfawzan (2018) used a combination of quantitative and qualitative methods to investigate FLA/FLE effects on FL achievement among FL students and found students with higher FLE had a lower level of FLA and a better academic performance. Moreover, it was emphasized that the positive correlation between FLE and academic performance was stronger than the negative correlation between FLA and academic performance. However, some scholars hold the opposite view, believing that FLA affected FL performance more than FLE. For example, Dewaele and Ergün (2020) investigated the correlation of FLA and FLE with academic performance of 110 Turkish middle school students learning English, and found that FLA was the strongest predictor of academic performance. Li (2020a) explored the relationship between students’ trait emotional intelligence, emotions and academic performance among 1,307 grade-two high school students in China, and found that the students with high FLE felt less FLA and had better learning performance, and that the influence of FLA on learning performance was more significant than that of FLE.

The above studies only confirm the predictive effect of FLA and FLE on general FL achievement. Dewaele and Li (2018) explored the correlation of FLA and FLE with achievements in specific FL skills (listening, speaking, reading, writing, vocabulary and grammar), suggesting that future research should integrate positive emotion regulation strategies into skill-specific interventions from the perspective of PP.

### Dynamicity of FLA and FLE

FLA and FLE are not completely opposed (Li and Han, 2022), mirroring PP’s dual-factor model of mental health that emphasizes the co-existence of distress reduction and well-being enhancement, and FLA and FLE are dynamic, so the relationship between them also changes dynamically (Dewaele and Dewaele, 2017), which deserves more dynamic research methods (Mierzwa, 2020). Boudreau et al. (2018) adopted dynamic systems theory to reveal the nonlinear interplay between FLA and FLE in second language communication, indicating that the dynamic relationship between FLA and FLE can be conceptualized and dynamically measured, which resonates with the emotion scaffolding concept in PP. By comparing the levels of FLA and FLE of the participants of different ages, Dewaele and Dewaele (2017) investigated how students’ FLA and FLE as well as their independent variables evolved over time. It was found that there was little change in FLA while FLE increased slightly, and that the prediction of different variables on FLA and FLE also varied with time. Aubrey (2022) adopted the idiodynamic approach and found that the negative relationship between them fluctuated from strong to very weak. Of note, Pan and Zhang (2021) adopted the longitudinal study method to unpack the changes of FLA and FLE in FL classroom over time, and found that FLE was less stable than FLA as time went on, which further validated the findings of Dewaele and Dewaele (2017).

## Conclusions and suggestions for future research

Although recent years witnessed more research on FLA and FLE since the introduction of PP into the field of language education, the research samples are mainly confined to college students and high school students. There is a scarcity of research focused on FLA and FLE of other student groups, such as FL beginner learners and FL learners with master degrees and PhD degrees. Meanwhile, FLA and FLE are different in different contexts. For example, Dewaele and MacIntyre (2014) found that the degree of FLA and FLE of Asian students was different from that of learners in other parts of the world. North American students experienced more FLE and less FLA, while Chinese students experienced a lower degree of FLE and a higher degree of FLA. This view is also supported by the findings of Jiang and Dewaele (2019) and Li (2020b). Besides, the levels of FLA and FLE of learners learning different FLs in the same country are also different. For example, in Belgium, English learners have significantly lower levels of FLA and higher levels of FLE than Dutch learners (De Smet et al., 2018). Therefore, in the light of PP, future studies on FLA and FLE should focus more attention on FL learners other than high school students and college students, FL learners in different contexts, and learners learning different FLs, especially non-common FLs.

With respect to research methods, most studies adopted quantitative research methods overwhelmingly represented by questionnaire survey. Future research on FLE and FLA should adopt both quantitative methods and qualitative methods to improve research reliability. Quantitative methods should employ context-based scales or questionnaires with good reliability and validity while qualitative methods can use classroom observation, video recording, student journals, field investigation, and particularly audio-recorded think-aloud protocols, instead of relying solely on semi-structured interviews. Kráľová et al. (2021) used qualitative research methods (questionnaire, interview and observation) and quantitative research methods (scale) to discuss which learning activities would lead to positive or negative emotions among high school students learning English as a FL. This study combined the four research methods into the research design, offering certain reference value for future study. Meanwhile, some advanced techniques like Event-related Potentials, Positron Emission Tomography and functional Magnetic Resonance Imaging can be incorporated into future research to uncover neural mechanism of FLE and FLA by measuring the electromagnetic, blood flow and neuronal activities of research samples.

Finally, most of the studies were cross-sectional studies, taking FLA and FLE as relatively stable individual variation variables. However, from the perspective of PP, FLA and FLE of FL learners are dynamic, and the influencing factors are complex and diverse. Diachronic studies can more scientifically reveal the dynamic changes of FLA and FLE. In the future, longer diachronic studies can be conducted on the dynamicity of FLA and FLE of learners of a certain FL, on the comparison of FLA and FLE among learners learning different FLs, and on the correlation of FLA and FLE with other variables.

## References

[ref1] Aida Y. (1994). Examination of Horwitz, Horwitz, and Cope’s construct of foreign language anxiety: the case of students of Japanese. Mod. Lang. J. 78, 155–168. doi: 10.1111/j.1540-4781.1994.tb02026.x, PMID: 40175095

[ref2] Ainley M. Hidi S. (2013). Interest and enjoyment. Int. Handbook Emotions Educ., eds. Pekrun R. Linnenbrink L. (New York: Routledge). 205–220. doi: 10.4324/9780203148211.ch11, PMID:

[ref3] Aubrey S. (2022). The relationship between anxiety, enjoyment, and breakdown fluency during second language speaking tasks: an idiodynamic investigation. Front. Psychol. 13:968946. doi: 10.3389/fpsyg.2022.968946, PMID: 36118455 PMC9472222

[ref4] Botes E. Dewaele J. M. Greiff S. (2021). Taking stock: an overview of the literature and a preliminary meta-analysis of foreign language enjoyment and other individual difference variables. PsyArXiv 1, 1–37. doi: 10.31234/osf.io/guaj5

[ref5] Boudreau C. MacIntyre P. D. Dewaele J. M. (2018). Enjoyment and anxiety in second language communication: an idiodynamic approach. Stud. Second Lang. Learn. Teach. 8, 149–170. doi: 10.14746/ssllt.2018.8.1.7

[ref6] Csikszentmihalyi M. (1990). Flow: the psychology of optimal experience. J. Leis. Res. 24, 93–94. doi: 10.1080/00222216.1992.11969876, PMID: 40101104

[ref7] De Smet A. Mettewie L. Galand B. Hiligsmann P. Van Mensel L. (2018). Classroom anxiety and enjoyment in CLIL and non-CLIL: does the target language matter? Stud. Second Lang. Learn. Teach. 8, 47–71. doi: 10.14746/ssllt.2018.8.1.3

[ref8] Dewaele J. M. (2013). The link between foreign language classroom anxiety and psychoticism, extraversion and neuroticism among adult bi- and multilinguals. Mod. Lang. J. 97, 670–684. doi: 10.1111/j.1540-4781.2013.12036.x, PMID: 40175095

[ref9] Dewaele J. M. (2022). “Enjoyment” in The Routledge handbook of second language acquisition and individual difference. eds. Li S. Hiver P. Papi M. (New York: Routledge), 190–206.

[ref10] Dewaele J. M. Alfawzan M. (2018). Does the effect of enjoyment outweight that of anxiety in foreign language performance? Stud. Second Lang. Learn. Teach. 8, 21–45. doi: 10.14746/ssllt.2018.8.1.2

[ref11] Dewaele J. M. Dewaele L. (2017). The dynamic interactions in foreign language classroom anxiety and foreign language enjoyment of pupils aged 12 to 18. A Pseudo-longitudinal investigation. J. Eur. Second Lang. Assoc. 1, 12–22. doi: 10.22599/jesla.6

[ref12] Dewaele J. M. Dewaele L. (2020). Are foreign language learners’ enjoyment and anxiety specific to the teacher? An investigation into the dynamics of learners’ classroom emotions. Stud. Second Lang. Learn. Teach. 10, 45–65. doi: 10.14746/ssllt.2020.10.1.3

[ref13] Dewaele J. M. Ergün A. L. P. (2020). How different are the relations between enjoyment, anxiety, attitudes/motivation and course marks in pupils’ Italian and English as foreign languages? J. Eur. Second Lang. Assoc. 4, 45–57. doi: 10.22599/jesla.65, PMID: 36447820

[ref14] Dewaele J. M. Li C. C. (2018). Emotions in SLA. Stud. Second Lang. Learn. Teach. 8, 15–19. doi: 10.14746/ssllt.2018.8.1.1

[ref15] Dewaele J. M. MacIntyre P. D. (2014). The two faces of Janus? Anxiety and enjoyment in the foreign language classroom. Stud. Second Lang. Learn. Teach. 4, 237–274. doi: 10.14746/ssllt.2014.4.2.5

[ref16] Dewaele J. M. MacIntyre P. D. (2016). “Foreign language enjoyment and foreign language classroom anxiety. The right and left feet of FL learning?” in Positive psychology in SLA. eds. MacIntyre P. Gregersen T. Mercer S. (Bristol: Multilingual Matters), 215–236. doi: 10.21832/9781783095360-010

[ref17] Dewaele J. M. MacIntyre P. D. (2019). “The predictive power of multicultural personality traits, learner and teacher variables on foreign language enjoyment and anxiety” in Evidence-based second language pedagogy: A collection of instructed second language acquisition studies. eds. Sato M. Loewen S. (London: Routledge), 263–286.

[ref18] Dewaele J. M. MacIntyre P. D. Boudreau C. Dewaele L. (2016). Do girls have all the fun? Anxiety and enjoyment in the foreign language classroom. Theory Practice Second Language Acquisition 1, 41–63.

[ref19] Dewaele J. M. Magdalena A. F. Saito K. (2019a). The effect of perception of teacher characteristics on Spanish EFL learners’ anxiety and enjoyment. Mod. Lang. J. 103, 412–427. doi: 10.1111/modl.12555, PMID: 40178379

[ref20] Dewaele J. M. Özdemir C. Karci D. Uysal S. Özdemir E. D. Balta N. (2019b). How distinctive is the foreign language enjoyment and foreign language classroom anxiety of Kazakh learners of Turkish? Applied Linguistics Review 13, 243–265. doi: 10.1515/applirev-2019-0021, PMID: 40160388

[ref21] Dewaele J. M. Witney J. Saito K. Dewaele L. (2017). Foreign language enjoyment and anxiety: the effect of teacher and learner variables. Lang. Teach. Res. 22, 676–697. doi: 10.1177/1362168817692161, PMID: 40160997

[ref22] Dewey D. P. Belnap R. K. Steffen P. (2018). Anxiety: stress, foreign language classroom anxiety, and enjoyment during study abroad in Amman, Jordan. Annu. Rev. Appl. Linguist. 38, 140–161. doi: 10.1017/S0267190518000107, PMID: 40166671

[ref23] Dong L. (2021). A meta-analysis on Chinese EFL learners’ foreign language anxiety and foreign language learning outcomes. Foreign Language World 1, 54–61.

[ref24] Du X. (2019). The revalidation of the foreign language classroom anxiety scale (FLCAS). Proceed. 2nd Int. Conf. Humanities Educ. Soc. Sci. Dordrecht: Atlantis Press. 626–631. doi: 10.2991/ichess-19.2019.130

[ref25] Elkhafaifi H. (2005). Listening comprehension and anxiety in the Arabic language classroom. Mod. Lang. J. 89, 206–220. doi: 10.1111/j.1540-4781.2005.00275.x, PMID: 40175095

[ref26] Fang F. Tang X. (2021). The relationship between Chinese English major students’ learning anxiety and enjoyment in an English language classroom: a positive psychology perspective. Front. Psychol. 12:705244. doi: 10.3389/fpsyg.2021.705244, PMID: 34335420 PMC8322688

[ref27] Gardner R. C. (1985). Social psychology and second language learning: The role of attitudes and motivation. London: Edward Arnold.

[ref28] Gardner R. C. MacIntyre P. D. (1993). On the measurement of affective variables in second language learning. Lang. Learn. 43, 157–194. doi: 10.1111/j.1467-1770.1992.tb00714.x, PMID: 40175095

[ref29] Garland E. L. Fredrickson B. Kring A. M. Johnson D. P. Meyer P. S. Penn D. L. (2010). Upward spirals of positive emotions counter downward spirals of negativity: insights from the broaden-and-build theory and affective neuroscience on the treatment of emotion dysfunctions and deficits in psychopathology. Clin. Psychol. Rev. 30, 849–864. doi: 10.1016/j.cpr.2010.03.002, PMID: 20363063 PMC2908186

[ref31] Gregersen T. MacIntyre P. D. (2014). Capitalizing on language learners’ individuality: from premise to practice. Bristol: Multilingual Matters. doi: 10.21832/9781783091218

[ref32] Horwitz E. K. (2001). Language anxiety and achievement. Annu. Rev. Appl. Linguist. 21, 112–126. doi: 10.1017/S0267190501000071

[ref33] Horwitz E. K. (2010). Foreign and second language anxiety. Lang. Teach. 43, 154–167. doi: 10.1017/S026144480999036X, PMID: 40166671

[ref34] Horwitz E. K. (2016). Factor structure of the foreign language classroom anxiety scale: comment on park (2014). Psychol. Rep. 119, 71–76. doi: 10.1177/0033294116653368, PMID: 27287268

[ref35] Horwitz E. K. Horwitz M. B. Cope J. (1986). Foreign language classroom anxiety. Mod. Lang. J. 70, 125–132. doi: 10.1111/j.1540-4781.1986.tb05256.x, PMID: 40178379

[ref36] Jiang Y. (2020). An investigation of the effect of teacher on Chinese university students’ foreign language enjoyment. Foreign Language World 1, 60–68.

[ref37] Jiang Y. Dewaele J. M. (2019). How unique is the foreign language classroom enjoyment and anxiety of Chinese EFL learners? System 82, 13–25. doi: 10.1016/j.system.2019.02.017

[ref38] Jin Y. Zhang L. J. (2018). The dimensions of foreign language classroom enjoyment and their effect on foreign language achievement. Int. J. Biling. Educ. Biling. 24, 948–962. doi: 10.1080/13670050.2018.1526253, PMID: 40101104

[ref39] Jin Y. Zhang L. J. (2019). A comparative study of two scales for foreign language classroom enjoyment. Percept. Mot. Skills 126, 1024–1041. doi: 10.1177/0031512519864471, PMID: 31345113

[ref40] Khajavy G. H. MacIntyre P. D. Barabadi E. (2017). Role of the emotions and classroom environment in willingness to communicate. Stud. Second. Lang. Acquis. 40, 605–624. doi: 10.1017/S0272263117000304, PMID: 40166671

[ref41] Kráľová Z. Kovacikova E. Repova V. Škorvagová E. (2021). Activities in English classes inducing positive/negative emotions. Educ. Sci. J. 23, 136–155. doi: 10.17853/1994-5639-2021-1-136-155

[ref42] Li C. (2020a). A positive psychology perspective on Chinese EFL students’ trait emotional intelligence, foreign language enjoyment and EFL learning achievement. J. Multiling. Multicult. Dev. 41, 246–263. doi: 10.1080/01434632.2019.1614187, PMID: 40101104

[ref43] Li C. (2020b). Emotional intelligence and English achievement: the mediating effects of enjoyment, anxiety and burnout. Foreign Language World 1, 69–78.

[ref44] Li C. Dewaele J. M. Jiang G. (2020). The complex relationship between classroom emotions and EFL achievement in China. Applied Linguistics Review 11, 485–510. doi: 10.1515/applirev-2018-0043, PMID: 40160388

[ref45] Li C. Han Y. (2022). The predictive effects of foreign language enjoyment, anxiety, and boredom on learning outcomes in online English classrooms. Modern Foreign Languages 2, 207–219. 10.3389/fpsyg.2022.1050226 PMC9831005 36636665

[ref46] Li C. Huang J. Li B. (2021). The predictive effects of classroom environment and trait emotional intelligence on foreign language enjoyment and anxiety. System 96:102393. doi: 10.1016/j.system.2020.102393, PMID: 40177572

[ref47] Li C. Jiang G. Dewaele J. M. (2018). Understanding Chinese high school students’ foreign language enjoyment: validation of the Chinese version of the foreign language enjoyment scale. System 76, 183–196. doi: 10.1016/j.system.2018.06.004

[ref48] MacIntyre P. D. (2017). “An overview of language anxiety research and trends in its development” in New insights into language anxiety: Theory, research and educational implications. eds. Gkonou C. Daubney M. Dewaele J. M. (Bristol: Multilingual Matters), 11–30.

[ref49] MacIntyre P. D. Gregersen T. (2012). Emotions that facilitate language learning: the positive broadening power of the imagination. Stud. Second Lang. Learn. Teach. 2, 193–213. doi: 10.14746/ssllt.2012.2.2.4

[ref50] Maican M. A. Cocorada E. (2021). Online foreign language learning in higher education and its correlates during the COVID-19 pandemic. Sustain. For. 13:781. doi: 10.3390/su13020781

[ref51] Mercer S. MacIntyre P. D. (2014). Introducing positive psychology to SLA. Stud. Second Lang. Learn. Teach. 4, 153–172. doi: 10.14746/ssllt.2014.4.2.2, PMID: 32982876

[ref52] Mierzwa E. (2020). Anxiety and enjoyment in the foreign language classroom. East West Cultural Passage 19, 107–121. doi: 10.2478/ewcp-2019-0007, PMID: 39513035

[ref53] Moskowitz S. Dewaele J. M. (2019). Is teacher happiness contagious? A study of the link between perceptions of language teacher happiness and student attitudes. Innov. Lang. Learn. Teach. 15, 117–130. doi: 10.1080/17501229.2019.1707205, PMID: 40101104

[ref54] Ohman A. (2008). “Fear and anxiety” in Handbook of emotions. eds. Lewis M. Haviland-Jones J. M. Barrett L. F.. 3rd ed (New York: Guilford), 709–729.

[ref55] Oxford R. L. (2017, 2017). “Anxious language learners can change their minds: ideas and strategies from traditional psychology and positive psychology” in New insights into language anxiety: Theory, research and educational implications. eds. Gkonou C. Daubney M. Dewaele J. M. (Bristol: Multilingual Matters), 177–197.

[ref56] Özer Z. Altay İ. F. (2021). Examining the level of enjoyment and anxiety among Turkish EFL students. J. Lang. Linguistic Studies 17, 663–671. doi: 10.17263/jlls.903532

[ref57] Pan C. Zhang X. (2021). A longitudinal study of foreign language anxiety and enjoyment. Lang. Teach. Res. 27, 1552–1575. doi: 10.1177/1362168821993341, PMID: 40160997

[ref58] Piechurska-Kuciel E. (2017). “L2 or L3? Foreign language enjoyment and proficiency” in Multiculturalism, multilingualism and the self: Studies in linguistics and language Learning. eds. Gabryś-Barker D. Gałajda D. (Spring: Cham), 97–111.

[ref59] Saito K. Dewaele J. M. Abe M. (2018). Motivation, emotion, learning experience and second language comprehensibility development in classroom settings: a cross-sectional and longitudinal study. Lang. Learn. 68, 709–743. doi: 10.1111/lang.12297

[ref60] Scovel T. (1978). The effect of affect on foreign language learning: a review of the anxiety research. Lang. Learn. 28, 129–142. doi: 10.1111/j.1467-1770.1978.tb00309.x, PMID: 40178379

[ref61] Seligman M. (2012). Flourish: A visionary new understanding of happiness and well-being. New York: Atria Books.

[ref62] Seligman M. Csikszentmihalyi M. (2000). Positive psychology: an introduction. Am. Psychol. 55, 5–14. doi: 10.1037/0003-066X.55.1.5 11392865

[ref63] Shirvan M. E. Taherian T. (2021). Longitudinal examination of university students’ foreign language enjoyment and foreign language classroom anxiety in the course of general English: latent growth curve modeling. Int. J. Biling. Educ. Biling. 24, 31–49. doi: 10.1080/13670050.2018.1441804, PMID: 40101104

[ref64] Spielberger C. D. Gorsuch R. Lushene R. E. Vagg P. R. (1983). Manual for the state-trait anxiety inventory (form Y1 – Y2). Palo Alto: Consulting Psychogyists Press.

[ref65] Wang C. K. (2003). The adaptation and validation of the foreign language classroom anxiety scale when applied to Chinese college students. Psychol. Sci. 2, 281–284.

[ref66] Wang Z. (2023). Psychological factors and production behaviors of Chinese undergraduate EFL learners. PLoS One 18:e0288014. doi: 10.1371/journal.pone.0288014, PMID: 37428752 PMC10332617

[ref67] Wen C. Liu G. Li J. (2021). An empirical study of Chinese L2 learners’ foreign language enjoyment and anxiety. J. Yunnan Normal Univ. 1, 44–53. doi: 10.16802/j.cnki.ynsddw.2021.01.006

[ref68] Zhao W. Zhang X. (2021). A study of foreign language anxiety and enjoyment of English major college students. Modern Commun. 7, 169–173.

